# Surface Topographic Features after Milling of Additively Manufactured AlSi10Mg Aluminum Alloy

**DOI:** 10.3390/ma15103604

**Published:** 2022-05-18

**Authors:** Grzegorz Struzikiewicz, Andrzej Sioma

**Affiliations:** Faculty of Mechanical Engineering and Robotics, AGH University of Science and Technology, 30-059 Kraków, Poland; andrzej.sioma@agh.edu.pl

**Keywords:** machining, sintered aluminum, 3D surface roughness parameters

## Abstract

The article presents selected issues related to material quality manufactured by selective laser sintering of AlSi10Mg alloy powder after milling. The workpiece was prepared and machined by down-milling and up-milling with tools made of high-speed steel. Breaches, pores and failure-like cracks on the machined surface were found, which negatively influenced the values of 3D surface roughness parameters. The occurring phenomena were analyzed and proposals for their explanation were made. The results of this research describe the effect of cutting parameters (the feed rate of *f* = 0.013–0.05 mm/tooth) on the values of parameters describing the surface quality and benchmarks. Topography measurements and 3D surface roughness parameters are presented, as well as the results of microscopic surface analysis. It was found that for aluminum alloy produced by the direct metal laser sintering (DMLS) method, the recommended machining method is down-milling.

## 1. Introduction

Currently, one of the trends in the manufacturing of metal machine parts is hybrid machining, which involves combining additive manufacturing (AM) technology with subtractive finishing [1]. Manufacturing parts by selective laser sintering (SLS) of metal powder with the ‘layer by layer’ method offers new possibilities for manufacturing. Unfortunately, additive manufacturing methods also have some disadvantages such as increased porosity, incomplete powder melting, insufficient dimensional and shape accuracy and high surface roughness [2,3]. Manufacturing parts in large quantities or with large dimensions can also be problematic due to cost and process efficiency. Therefore, research is being conducted to increase the quality and properties of parts obtained by additive methods through the appropriate selection and optimization of 3D printing process parameters. Hybrid machining is another way to improve the dimension and shape accuracy and surface quality. In this sense, this machining consists of pre-fabricating a part using additive methods (e.g., DMLS), and then finally giving it the required accuracy using subtractive machining such as chip machining or electrical discharge machining [4,5].

Most often, authors of scientific publications address issues related to improving the quality of parts produced by AM. This includes an analysis of the impact of the 3D printing process parameters on the quality of manufactured parts. The energy density of the laser beam is the basic parameter under analysis. The findings of the work of Baitz et al. [6] and Bai et al. [7] show that the laser beam energy density increases along with increased laser power, decrease in scanning speed and powder layer thickness. As the energy density of the laser beam increases, the density of parts made by the selective laser melting (SLM) process initially increases and then decreases. An increase in the energy density of the laser beam causes an increase in temperature, resulting in a large amount of low-viscosity liquid metal that flows easily and fills the pores. If the energy density of the laser beam is too high, it contributes to the accelerated evaporation of the molten material and the formation of spherical pores, as described by Khairallah et al. [8]. At low laser energy consumption, large and irregular pores can form, as shown by Kabir et al. [9]. The surface roughness along with the porosity of SLM-fabricated parts initially decreases and then increases along with increasing the energy density of the laser beam [10].

In the literature, many authors are involved in experimental studies to optimize the parameters of the SLM process and the powder melting of the AlSi10Mg aluminum alloy. The aim of this research is to obtain manufactured parts with high density, low surface porosity and roughness. Wei et al. [11] and Wang et al. [12] analyzed the effect of the scanning rate and laser power. The results showed that more pores and also numerous defects in the material microstructure and undissolved metal powder particles are formed when high scanning rates are used. On the other hand, Maamoun et al. [13] obtained the higher material density by combining the higher scanning rate and the laser power or by combining a lower scanning rate and moderate laser power. The authors concluded that the optimal laser beam energy density range to melt AlSi10Mg powder is 50–60 J/mm^3^, as this minimizes the occurrence of pores in the structure. Similar conclusions were reached by Trevisan et al. [14] and Gao et al. [15].

There are not many publications in the literature on the machining of parts produced by laser sintering of metal powder. In this field, the analysis of the effect of cutting parameters on the roughness of the machined surface is the most common topic. For example, Kaynak and Kitay [16] and also Struzikiewicz et al. [1] used turning to reduce the surface roughness of a part fabricated from stainless steel. They concluded that the feed rate of the cutting tool has the greatest effect on surface roughness. Analogous results were obtained by the authors Yassin [17] after milling a part produced by the SLM method from CM-Ni-Cu powder. However, the authors of this paper did not analyze the effect of the SLM process parameters. This type of problem was analyzed by Milton et al. [18] who investigated the effect of the orientation of the SLM machine coordinate system when manufacturing parts from alloy powders of Ti6Al4V. The authors examined the surface roughness obtained after milling and found that the lowest roughness was obtained after milling in the X–Z plane and the highest in the X–Y plane.

Other studies have considered the machinability of materials manufactured by the SLM process compared to those produced conventionally. Struzikiewicz et al. [19] analyzed the turning of aluminum alloy and noticed many deformations on the machined surface. During machining with different cutting parameters, a reduction in the surface roughness values was obtained for the comparison material produced by casting. Dumas et al. [20], on the other hand, did not see significant differences between SLM and traditionally manufactured Ti6Al4V alloy.

Evaluation of surface topography after machining is important for its functionality in further applications. The properties of the material in contact, surface load-carrying ability, friction, lubricant retention and wear resistance, are all related to surface topography. Therefore, the results of surface topography measurements are important to both manufacturers and tribologists. The 3D (real) surface parameters are more reliable than the 2D profile parameters. On the other hand, 2D parameters like *Ra* or *Rz* are often used in industry. Other parameters, such as spatial, hybrid or functional ones, can deliver more information about the surface [21].

The maximum height is considered to be associated with surface damage and the averaged parameters with normal surface operation [22]. Parameters such as *Sq*, *Sa*, *Sp*, *Sv* and *Sz* characterize the surface amplitude, while *Ssk* and *Sku* describe the nature of the height distribution. The parameters *Sq* and *Sa* are similar (usually slightly higher) to the roughness parameters *Rq* and *Ra* of isotropic surfaces, respectively.

In research work in tribology [23] and machining [24,25], various parameters written in ISO 25178-2 are considered. For example, lower values of the *Sa* and *Sq* parameters correspond to a higher surface gloss [24]. On the other hand, based on changes in the value of the *Sv* parameter, it is possible to obtain information about whether the wear or plastic strain of the displaced surfaces occurred before and after the tribological test. When the change in *Sv* is close to 0, plastic strain occurs [25]. 

The amplitude parameters are related to friction and wear. The types of friction depend on the height of the surface. Fluid friction occurs when the thickness of the oil film is greater than the sum of the heights of the two contacting surfaces. A smooth surface tends to seize up because of the difficulty in holding oil [26]. 

The parameters *Ssk* and *Sku* were used to identify the surface after different treatment processes. Świrad et al. [27] found that these parameters are sensitive to the burnishing parameters. Mezari et al. [28] obtained the relationship between different types of whetstones and skewness and kurtosis. Negative skewness is characteristic of multi-processed (layered) surfaces and porous materials. The results presented in [29] show that negative skewness and low kurtosis improve the contact of rough surfaces by increasing normal stiffness. 

Surface texturing is an option to improve the tribological properties of sliding elements as reported by Etsion [30] and Gachot et al. [31]. Dimples (pockets or oil recesses) lead to reduced friction resistance in mixed boundary and fluid lubrication. The presence of dimples can improve the seizure resistance of sliding assemblies. Oil pockets can also be traps for abrasive particles. There are many articles on friction reduction due to surface texturing under lubricated conditions. The presence of dimples can improve the tribological performance of seals, plain bearings or cylinder liners as reported by Sharma et al. [32] and Morris et al. [33]. In general, negatively angled surfaces have good grease retention. However, skewness cannot characterize completely textured surfaces. Other parameters such as dimple area ratio, dimple size and dimple volume are also important. The paper [34] presents methods for estimating oil capacity. For dual-process textures, two parameters are proposed to describe the amplitude: they should characterize the peak and valley parts. Fecske et al. [35] recommended the parameter *Sq* and skewness to characterize the texture height. Some of the other 3D parameters are related to the material surface coefficient curve and are called functional parameters. There are three families of parameters: the *Sk* group, the *V* group and the *Sq* group. The *Sk* family includes the following parameters: core height *Skv*, reduced peak height *Spk*, reduced dimple height *Svk* and material ratios *Sr1* and *Sr2*. Group *V* consists of the following parameters: core void volume *Vvv*, core void volume *Vvc*, peak material volume *Vmp* and core material volume *Vmc*. There are three parameters of the *Sq* family: *Spq*, *Smq*. The material ratio curve has many useful applications, such as determining the oil capacity, the dimple ratio of a textured surface, or assessing low wear. It can be applied not only to the surface of the cylinder liners but also to other textures, for example, after additive manufacturing [36].

Other parameters are surface feature parameters. According to ISO 25178 [37], these are parameters such as density of peaks *Spd*, arithmetical average peak curvature *Spc* or five-point pit height *S5v*.

The feature-based surface characterization technique has been applied in various areas, such as machining. Tian et al. [38] used feature parameters to evaluate the surface topography of the wear particle. Ye et al. [39] used a feature-based characterization technique to characterize the topography of a diamond grinding wheel. The watershed segmentation method is also suitable for analyzing additively generated surfaces of an arbitrary shape [40]. 

There is little knowledge of the functional significance of various surface properties. A review of the literature indicates that there are no publications that simultaneously analyze the effect of metal powder laser sintering process parameters and subsequent material processing on the obtained surface roughness. It is clear that simultaneous optimization at the manufacturing and material processing stages can benefit in the form of more optimized results. Therefore, this paper considers the simultaneous analysis and optimization of selected DMLS parameters and milling process parameters. The authors attempted to establish optimal milling parameters of laser-sintered AlSi10Mg alloy, taking into account the criterion of the machined surface quality described by 3D surface roughness parameters. These include height, space and hybrid and functional parameters. The analysis of down-milling and up-milling of sintered material and roughness, topography and microscopic measurements of the machined surface were carried out. The results presented in the paper are a continuation of the analyses undertaken by the authors as previously described [41].

## 2. Materials and Methods

In order to carry out the tests, a cutting sample was prepared. The workpiece was manufactured using Renishaw’s (Wotton-under-Edge, New Mills, UK) AM 250 system by selective laser sintering of AlSi10Mg aluminum powder. A workpiece with a circular cross-section of θ50 mm × 50 mm was manufactured. The chemical composition of the isolated material: 89.26% Al, 9.74% Si, 0.312% Fe, 0.44% Mn, 0.20% Mg, 0.11% Cu, <0.004% for other elements. The DMLS process parameters were chosen on the basis of the literature review. The machine was equipped with the YFL continuous wave Ytterbium fiber laser (wavelength of 1070 nm), allowing the sintering and melting of elements with a power of 400 W and a laser beam diameter equal to 70 μm. The laser scanning speed was variable in the 600–2000 mm/s range. The thickness of the powder layer was equal to 25 µm. The process was conducted in the protective argon atmosphere. The particle size distribution was 20–63 μm. The AlSi10Mg alloy is characterized by the following properties: tensile strength *Rm* = 193 MPa, elongation *A5* = 2.5% and Brinnel hardness 68HB. The density of the material after sintering was determined to be ρ = 0.064 g/mm^3^. The mechanical properties of the alloy make it feasible to use it in the manufacturing of large-size castings of complex shape and high strength, such as gearbox casings in motor vehicles, steering gear bodies in cars and internal combustion engine blocks in motor vehicles.

**Figure 1:**
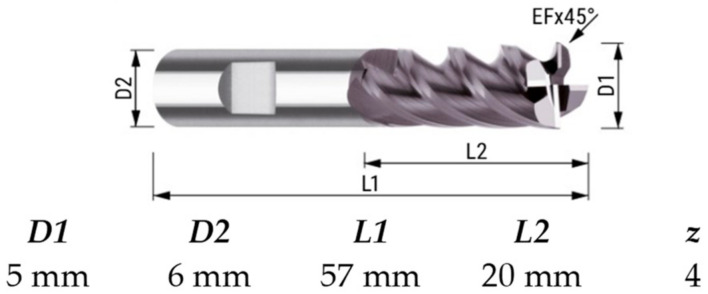
The geometry of the milling tool.

The milling was performed on a CNC MiniMill2 machine tool (Haas, Oxnard, CA, USA) using a 4-edged endmill cutter with a 5 mm diameter and catalogue number 224.050.00 (InovaTools, Kinding, Germany). The cutting tool was coated with a TiAlN coating. The machining parameters were chosen on the basis of the recommendations of the cutting tool manufacturer. The tool is characterized in Figure 1. Dry processing was used. The down and up milling processes were analyzed. A constant depth of cut *a_p_* = 1.0 mm and milling width *a_e_* = 5 mm were assumed. Cutting tests were conducted for feed rates in the range *f_z_* = 0.013; 0.017; 0.025; 0.05 mm/tooth.

During the tests, 3D surface topography and microscopic measurements of the machined surface were conducted. The measured parameters were achieved according to ISO 4287 and ISO 25178. Measurements were captured with profilographometer Talysurf Intra 50 manufactured by Taylor Hobson (Leicester, UK). The microscopic analysis of the machined surface was carried out using a 3D microscope VHX-7000 by Keyence (Osaka, Japan), with a resolution of 0.5 nanometers on the Z-axis and 130 nanometers on the XY axis. The imaging field was 705 microns on the X-axis and 528 microns on the Y-axis. 

The analysis of the influence of cutting parameters on the roughness of the surface and the accuracy of the dimension and shape of machined parts is often carried out on the basis of various methods, such as Taguchi [42]. The experimental research plan was developed according to the Taguchi method. The method was chosen because it can be used to optimize and design the properties of products and parameters of manufacturing processes resistant to various types of interference, both during the production of products and their operation. The influence of variable cutting parameters, i.e., feed per tooth *f_z_* and milling method-down or up-on values of 3D surface roughness parameters was analyzed. In the statistical analysis of the test results, the matching function model according to Formula (1) was adopted.

(1) $$Y_{1}=y-\varepsilon =b_{0}x_{0}+b_{1}x_{1}+b_{2}x_{2}+b_{3}x_{3}+b_{4}x_{4},$$

In Formula (1) *Y*_1_ is the estimated response based on the first order equation and *y* is the measured parameter (e.g., roughness parameter) on a logarithmic scale where *x*_0_ = 1 (dummy variable) and *x*_1_–*x*_4_ are the logarithmic transformations of parameters; ε is the experimental error and the values *b* are estimates of the corresponding parameters.

The S/N (signal-to-noise) ratio analysis strategy was adopted as “the lowest-the best” according to Formula (2). The level of significance α = 0.05 was adopted.

(2) $$S/N=-10\;\cdot \;log\left(\frac{1}{n}\overset{n}{\underset{i=1}{\sum }}y_{i}^{2}\right),$$

where *y_i_* is the respective characteristic and *n* is the number of observations.

## 3. Results and Discussion

**Figure 2:**
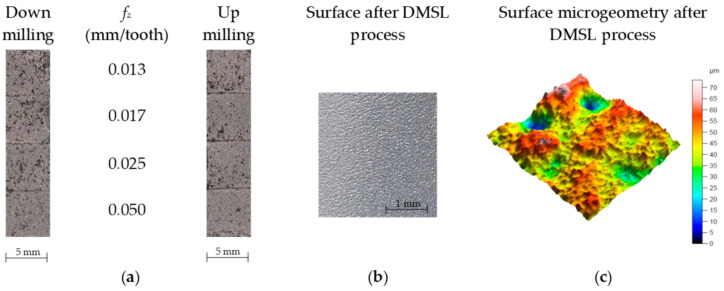
Surface after down and up milling (**a**) and before machining (**b**) including microgeometry (**c**).

According to the adopted test plan (Table 1), cutting tests were carried (i.e., down and up milling) of the workpiece made by direct metal laser sintering (DMLS). Microscopic observations and measurements of selected 3D parameters of surface roughness and machined surface characteristics were performed afterward. Figure 2 presents photographs of the surfaces obtained in the cutting tests.

**Figure 3:**
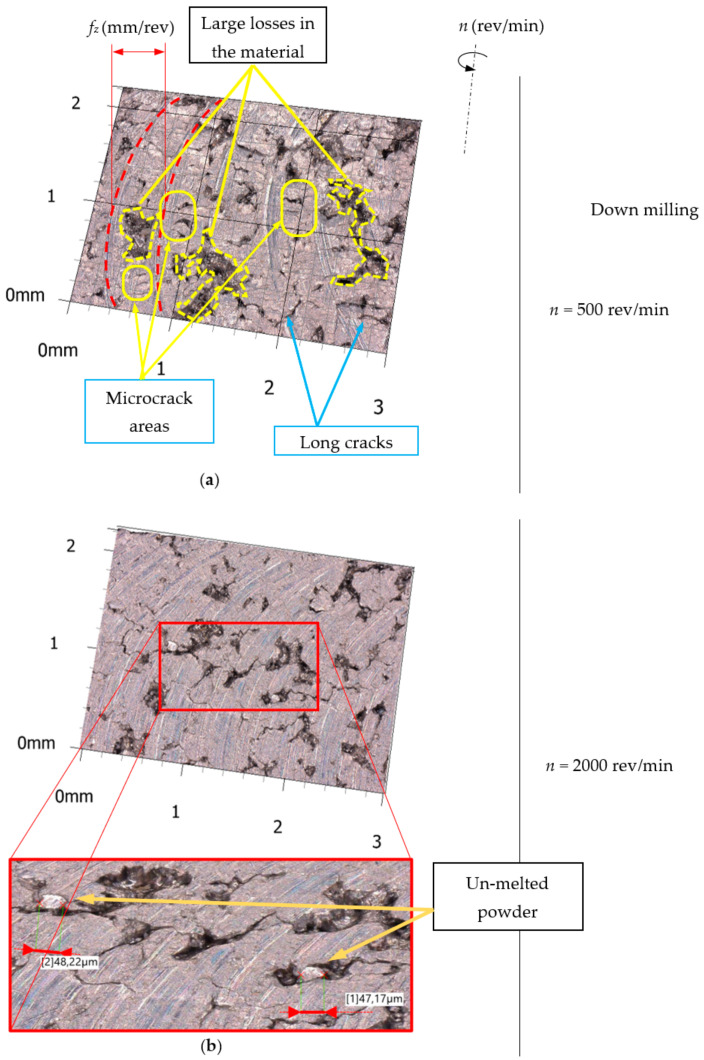
Example of 3D microscope surface measurement after down milling. (**a**) *n* = 500 rev/min. (**b**) *n* = 2000 rev/min.

**Figure 4:**
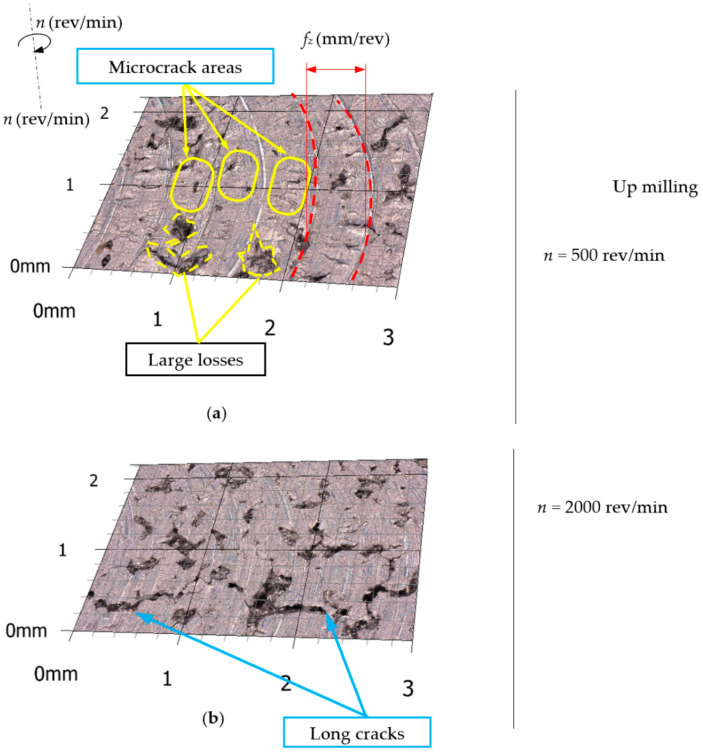
Example of 3D microscope surface measurement after up milling. (**a**) *n* = 500 rev/min. (**b**) *n* = 2000 rev/min.

The analysis of microscopic measurement results showed numerous breaches that occurred after milling the surface of the workpiece. Figure 3 presents example microscopic images of the aluminum alloy surface after down milling, and Figure 4 presents example images for up milling.

Microscopic analysis revealed numerous areas of microcracks at the bottom of the machining tracks after the passage of the cutting tool. Long cracks were also observed on the surface of the material arranged perpendicular to the machining tracks (especially for the feed rate *f_z_* = 0.05 mm/tooth). Moreover, microscopic observations of the machined surfaces showed numerous breaches from the machined surface. The distribution, number, area and depth of the breaches were random. Material breaches and cutting blade tracks on the machined surface can affect the results of 2D surface roughness parameter measurements. Therefore, the 3D surface roughness parameters were measured.

**Figure 5:**
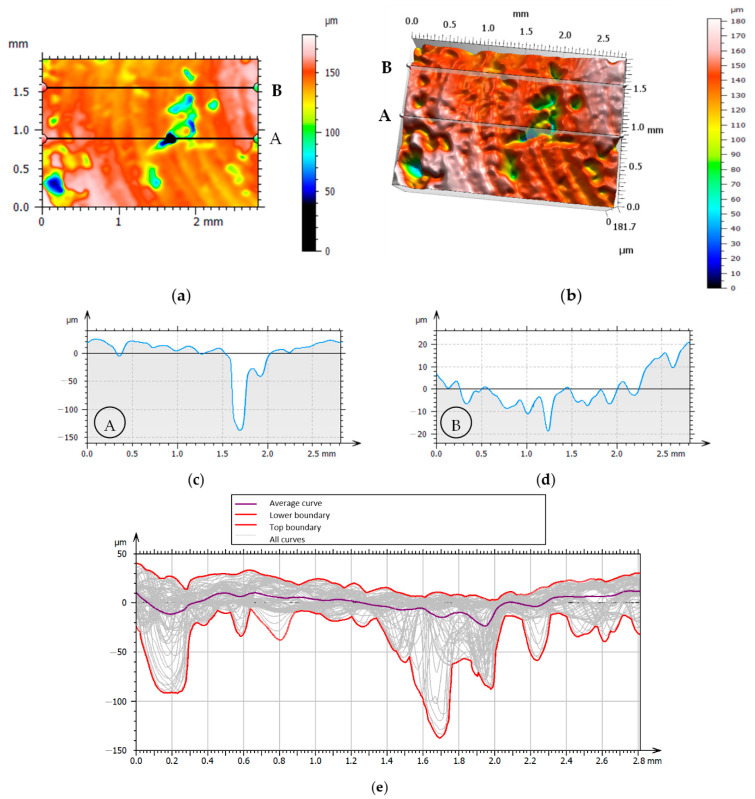
Examples of surface topography—test 5, (**a**) top view, (**b**) angled view, (**c**) profile A, (**d**) profile B, (**e**) collection of profiles.

Figure 5 presents selected topographies of the machined surface and surface profiles. Significant differences were observed in the results of 2D surface roughness parameter measurements due to the position of the measurement segment, e.g., positions A and B in Figure 5a,b. The obtained profile curves for positions A and B are shown in Figure 5c, and Figure 5c,d show the result of determining the average surface profile. Table 2 presents the results of 2D surface roughness parameter measurements for both cases.

The values obtained for the *Rp*, *Rv*, *Rz*, *Rt* and *Ra* parameters vary about 4–5 times depending on the position of the measurement line (in this case, lines A and B in Figure 5). 

**Figure 6:**
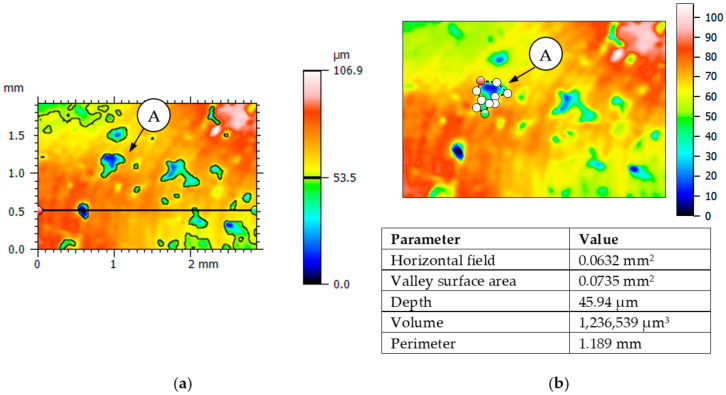
Examples of the surface topography analysis of test 1—location of the breaches (**a**) and measurement of the area and depth of the breach (**b**) (from 3D microscope).

An example analysis of the distribution and geometric dimensions for selected breaches is shown in Figure 6.

**Figure 7:**
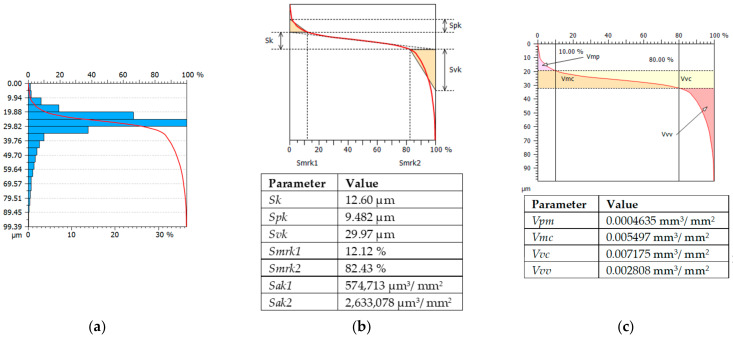
Examples of 3D measurements of surface parameters (**a**) Abott curve, height (**b**) and volume (**c**) test 1 surface topography.

Figure 7 presents an example histogram along with an Abott curve and an analysis of selected surface elevation and volume parameters.

Based on the analysis conducted to determine the effect of variables (feed rate and type of milling) on the surface quality, parameters related to and characterizing the breaches occurring on the surface were selected. Table 3 presents selected values of the 3D surface roughness parameters after milling. Comparatively, in Table 4 selected values of 3D surface roughness parameters are presented before treatment, i.e., obtained by laser sintering. The general comparison of the results indicates that the use of milling under the adopted conditions contributes to an increase in the values of the 3D parameters, which implies the deterioration of surface quality.

**Figure 8:**
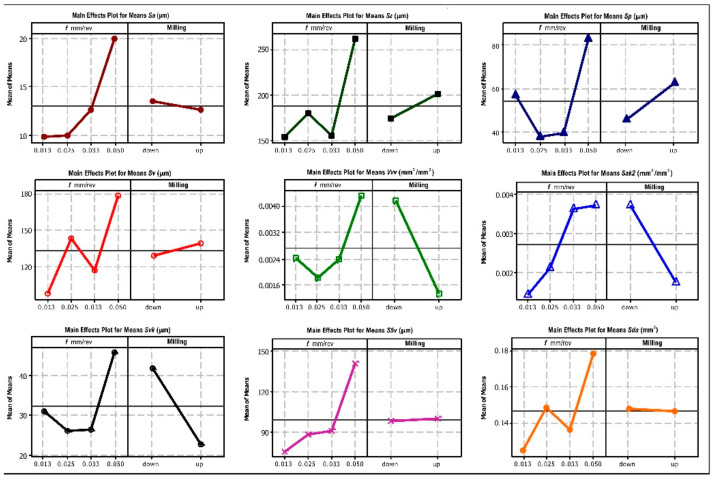
Influence of cutting data on the parameters *Sa* (μm), *Sz* (μm), *Sp* (μm), *Sv* (μm), *Vvv* (mm^3^/mm^2^), *Sak2* (mm^3^/mm^2^), *Svk* (μm), *S5v* (μm), *Sda* (mm^2^).

**Figure 9:**
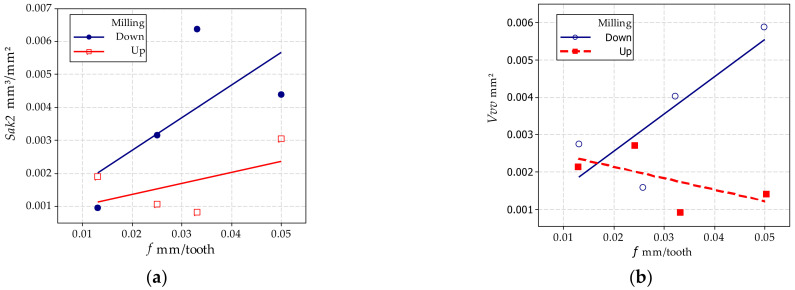
Graphical representation of the effect of feed rate and milling type on the parameters *Sak2* (**a**) and *Vvv* (**b**).

Analysis of the results showed that the variables (i.e., feed rate and type of milling) significantly affect the parameters that characterize the machined surface (Figure 8). In general, down-milling results in lower roughness parameters. The analysis shows that the values of all the parameters considered are significantly affected by the milling feed rate *f*. In turn, the effect of the type of milling (down or up) on the values of individual parameters can be variable. For example, for parameters *Sa* and *Sz*. This may be due to the fact that peak heights on the surface are included in the calculations and the parameter values are averaged. This can be confirmed by the values of the parameter *Sp*. On the other hand, the analysis of the parameters directly related to the breaches, i.e., their depth, area and volume, shows that there is no significant effect of the milling method (e.g., for parameters *S5v* and *Sda*). The parameters related to the volume of surface voids as *Vvv* and its *Sak2* surface show a different trend. Figure 9 presents the influence of the feed rate *f* for down and up milling on the values characterizing the machined surface, i.e., *Sak2* and the volume of breaches *Vvv*. The results indicate that increasing the feed rate of the cutting tool increases the values of the *Sak2* parameters and thus, the area of the breaches. In addition, the volume of breaches is significantly larger for the up-milling case (Figure 9b). For these cutting conditions, down-milling produces a better-machined surface.

The Table 5 shows the results of the ANOVA analysis of the components for the 3D parameters (where: DF—degrees of freedom. Seq SS—sums of squares. Adj SS—adjusted sums of squares. Adj MS—adjusted means squares).

Based on Table 5 we can state that the parameter *A*—the freed, has a statistically significant (at the assumed level α = 0.05) influence on the parameter *Sa*. In this case *p* = 0.017 < 0.05. On the other hand, for parameter *B*, i.e., the type of milling, the probability of *p* = 0.478 means that there is no significant effect on the values of the *Sa* parameter. Based on the data in the table, it may be assumed that the *Sa* parameter is of key importance in the process of evaluation of defects formed on the machined surface of the aluminum printed material. For the remaining parameters, the results show no significant effect of the analyzed parameters on the values of particular parameters. However, the observations and analyses that have been made previously allow for practical conclusions. The analysis shows that the most important parameter that influences the values of the 3D parameters is the feed rate *f*. For example, the lowest values of the parameters *Sa* and *Sz* were obtained for the feed rate *f* = 0.013 mm/tooth. Furthermore, analysis of the results shows that the type of milling has a variable influence on the 3D values of the parameters that characterize the surface roughness. Down milling most significantly affects the volumetric parameters, e.g., *Vvv* and *Svk* and also the parameter *Sak2*. On the other hand, the type of milling has the smallest effect on the parameters *Spv* and *Sda*.

**Figure 10:**
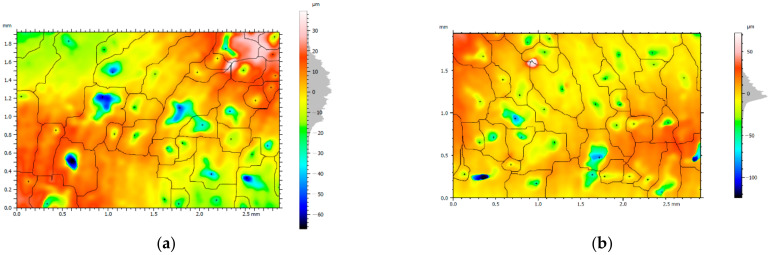

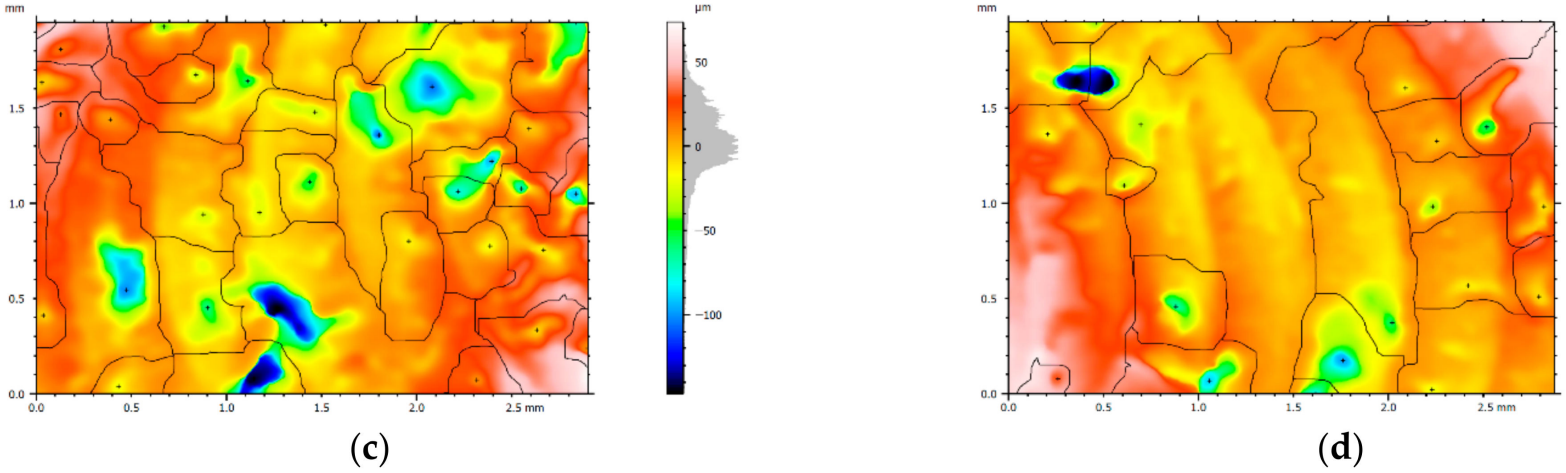
3D Watershed segmentation—(**a**) area for test 7 (*f* = 0.013 mm/tooth, down milling), particle count 40, density 7.228 particles/mm^2^ (**b**) area for test 8 (f = 0.013 mm/tooth, up milling), particle count 39, density 6.912 particles/mm^2^ (**c**) area for test 1 (*f* = 0.05 mm/tooth, down milling), particle count 34, density 6.014 particles/mm^2^ (**d**) area for test 2 (*f* = 0.05 mm/tooth, counter-rotating milling), particle count 20, density 3.569 particles/mm^2^.

In the next step, 3D watershed segmentation analysis of particle detection with height pruning was performed according to Wolf’s algorithm with 5% *Sz.* Example analyses are shown in Figure 10. The analysis demonstrated higher particle densities for decreasing values of feed rate *f*. A reduction in the number of particles was also observed for up milling.

Analysis of the research results showed that the number and geometric dimensions of cracks and breaches on the machined surface, i.e., their width and depth, depend on the value of cutting parameters. The depth, area and count of the breach are included in the values of 3D parameters (i.e., *Svk* and *Vvv*) that describe the surface quality. The occurrence of breaches on the surface after treatment may be related to the structure and properties of the successive layers of material formed during laser sintering, which depends on the remelting conditions of the metal powder and is characterized by porosity as described, for example, by Kempen et al. [43] and defects (cracks) as reported by Read et al. [44]. This can cause the cutting tool to pull material particles from the workpiece surface during operation. The pressure of the cutting edge on the workpiece material can cause microcracks to form which can then develop into larger cracks. Cracks in the material can be reinforced along the melt line of the material. The presence of porous surfaces present in materials obtained by laser sintering may also be an additional factor. The cause of breaches on the machined surface is probably the method and conditions of combining material particles during the laser sintering. The process carried out in this way involves melting, then cooling and fusing the metal powder particles. Consequently, the structure created from the material is characterized by the presence of areas of weaker bonding of the material particles. In addition, the cohesion forces of the material particles may be lower due to the incomplete melting of the metal powder by the laser beam. Therefore, at the applied feed rate of the cutting tool, conditions are created in the decohesion zone of the material that are conducive to plastic strain and removal of particles from the workpiece material.

## 4. Conclusions

Based on the results obtained and the analyses carried out, the following conclusions, regarding the development of breaches formed during the milling of AlSi10MG aluminum parts made by the DMLS method, can be drawn:

The surface quality after milling under the adopted cutting conditions is inferior to that obtained after selective laser sintering. This is caused by the formation of deep and superficial breaches on the surface after machining, as well as clear tracks after the passage of the cutting edge.

Interpreting the results obtained results of 3D parameters performed separately (for a single parameter) can be difficult. A better way to determine surface quality is to measure several parameters that describe surface features and then interpret them together. For example, the values of parameters *Svk*, *Vvv* contain more useful information on the surface quality than 2D parameters (e.g., *Ra*, *Rz*) and 3D such as *Sa* and *Sz*.

After milling, there are numerous material breaches on the surface of sintered aluminum. The distribution of breaches, their area and depths is uneven. The number and geometric dimensions of breaches on a machined surface can affect the operational properties of the workpiece (e.g., surface load-carrying ability). The depth and size of breaches are determined by the feed rate of the cutting edge and the milling method. The maximum depths of the breaches were over 150 μm. The down-milling has a positive effect on the quality of the machined surface. 

## Figures and Tables

**Table 1:** Research plan with real values.

No.	A	B	*f* (mm/Tooth)	Milling
1	1	1	0.050	down
2	1	2	0.050	up
3	2	1	0.033	down
4	2	2	0.033	up
5	3	1	0.025	down
6	3	2	0.025	up
7	4	1	0.013	down
8	4	2	0.013	up

**Table 2:** Measurement results of the roughness parameters (ISO4287) profiles A and B in Figure 5.

Profile A			Profile B		
*Rp*	21.68	µm	*Rp*	4.37	µm
*Rv*	33.69	µm	*Rv*	7.39	µm
*Rz*	55.37	µm	*Rz*	11.76	µm
*Rt*	112.0	µm	*Rt*	16.47	µm
*Ra*	10.92	µm	*Ra*	2.49	µm

**Table 3:** Summary of measurement results for 3D parameters.

No.	f	Milling	Sa	Sz	Sq	Sku	Sp	Sv	Sk	Spk	Svk	Smrk1	Smrk2	Sak1	Sak2	Vmp	Vmc	Vvc	Vvv	Spd	Spc	S5v	Sda	Sdv
	mm/ tooth		µm	µm	µm	µm	µm	µm	µm	µm	µm	%	%	mm^3^/mm^2^	mm^3^/mm^2^	mm^3^/mm^2^	mm^3^/mm^2^	mm^3^/mm^2^	mm^3^/mm^2^	1/ mm^2^	1/mm	µm	mm^2^	mm^3^
1	0.050	Down	19.16	220.51	27.96	7.61	73.63	146.88	46.59	18.92	63.14	13.39	86.13	0.0013	0.0044	0.0009	0.0172	0.0246	0.0057	2.12	26.47	120.56	0.150	0.00033
2	0.050	Up	16.25	235.77	24.34	11.03	71.98	163.79	34.69	34.09	48.67	20.72	90.15	0.0035	0.0024	0.0012	0.0157	0.0290	0.0032	1.61	27.25	125.58	0.162	0.00043
3	0.033	Down	13.94	189.10	21.55	14.40	29.31	159.79	18.69	4.61	51.75	6.19	75.35	0.0001	0.0064	0.0003	0.0118	0.0098	0.0054	2.38	47.58	118.85	0.150	0.00052
4	0.033	Up	11.44	123.38	14.89	3.98	49.68	73.70	27.39	20.14	20.40	21.74	91.70	0.0022	0.0008	0.0007	0.0115	0.0206	0.0016	3.00	22.24	63.58	0.125	0.00022
5	0.025	Down	11.35	181.66	18.02	13.21	40.26	141.40	23.79	11.11	44.00	17.78	85.50	0.0010	0.0032	0.0004	0.0097	0.0144	0.0038	1.85	23.97	95.99	0.168	0.00051
6	0.025	Up	8.55	179.07	12.98	28.18	34.81	144.25	25.38	10.11	31.88	11.33	93.25	0.0006	0.0011	0.0005	0.0088	0.0124	0.0017	0.73	297.30	80.79	0.129	0.00052
7	0.013	Down	9.61	106.90	12.51	5.07	39.73	67.17	30.47	10.15	19.19	6.49	89.94	0.0003	0.0010	0.0005	0.0109	0.0132	0.0017	1.45	14.09	56.58	0.124	0.00031
8	0.013	Up	9.75	196.23	14.71	12.94	71.85	124.38	23.24	13.83	33.06	16.70	88.77	0.0012	0.0019	0.0006	0.0087	0.0142	0.0025	1.24	139.77	92.81	0.123	0.00024

**Table 4:** Selected values of surface roughness parameters after laser sintering.

Parameter	Result 1	Result 2	Result 3	Average Value
*Sq* µm	2.77	3.47	3.12	3.12
*Ssk*	−0.285	−0.0392	−0.159	−0.161
*Sku*	3.95	3.51	4.14	3.87
*Sp* µm	9.4	13.3	16	12.9
*Sv* µm	16.6	14.3	18	16.3
*Sz* µm	26	27.6	34	29.2
*Sa* µm	2.16	2.7	2.42	2.43

**Table 5:** Analysis of Variance for Means.

Source	DF	Seq SS	Adj SS	Adj MS	F	P	Seq SS	Adj SS	Adj MS	F	P
		*Sa* (µm)					*Sz* (µm)				
A	3	137.18	137.18	45.73	20.5	0.017	15231	15231	5077	1.79	0.322
B	1	1.46	1.46	1.46	0.65	0.478	1418	1418	1418	0.5	0.53
Residual Error	3	6.69	6.69	2.23			8489	8489	2830		
Total	7	145.34					25138				
		*Sp* (µm)					*Sv* (µm)				
A	3	2664.9	2664.9	888.3	6.85	0.074	7379.1	7379.1	2459.7	1.01	0.498
B	1	555.5	555.5	555.5	4.28	0.13	198.6	198.6	198.6	0.08	0.794
Residual Error	3	389	389	129.7			7338.4	7338.4	2446.1		
Total	7	3609.4					14916				
		*Vvv* (mm^3^/mm^2^)					*Sak2* (mm^3^/mm^2^)				
A	3	0.000003	0.000003	0.000001	0.27	0.846	0.000008	0.000008	0.000003	0.7	0.61
B	1	0.000006	0.000006	0.000006	1.83	0.269	0.000008	0.000008	0.000008	2.23	0.232
Residual Error	3	0.000010	0.00001	0.000003			0.000011	0.000011	0.000004		
Total	7	0.000019					0.000027				
		*Svk* (µm)					*S5v* (µm)				
A	3	519.2	519.2	173.1	0.42	0.751	4901.99	4901.99	1634	1.54	0.366
B	1	731.7	731.7	731.7	1.79	0.273	7.57	7.57	7.57	0.01	0.938
Residual Error	3	1226.5	1226.5	408.8			3182.79	3182.79	1060.93		
Total	7	2477.4					8092.35				
		*Sda* (mm^2^)									
A	3	0.0032	0.0032	0.0011	1.26	0.428					
B	1	0.0000	0.0000	0.0000	0	0.959					
Residual Error	3	0.0025	0.0025	0.0008							
Total	7	0.0057									

## Data Availability

The data presented in this study are available on request from the corresponding author. The data are not publicly available due to privacy.
